# Neurologically Potent Molecules from *Crataegus oxyacantha*; Isolation, Anticholinesterase Inhibition, and Molecular Docking

**DOI:** 10.3389/fphar.2017.00327

**Published:** 2017-06-07

**Authors:** Mumtaz Ali, Sultan Muhammad, Muhammad R. Shah, Ajmal Khan, Umer Rashid, Umar Farooq, Farhat Ullah, Abdul Sadiq, Muhammad Ayaz, Majid Ali, Manzoor Ahmad, Abdul Latif

**Affiliations:** ^1^Department of Chemistry, University of MalakandChakdara, Pakistan; ^2^International Center for Chemical and Biological Sciences, University of KarachiKarachi, Pakistan; ^3^COMSATS Institute of Information TechnologyAbbottabad, Pakistan; ^4^UoN Chair of Oman Medicinal Plants and Marine Products, University of NizwaNizwa, Oman; ^5^Department of Pharmacy, University of MalakandChakdara, Pakistan

**Keywords:** *Crataegus oxyacantha*, Alzheimer’s disease, acetylcholinesterase (AChE) inhibition, butyrylcholinesterase (BChE) inhibition, molecular docking, pharmacokinetic properties

## Abstract

*Crataegus oxyacantha* is an important herbal supplement and famous for its antioxidant potential. The antioxidant in combination with anticholinesterase activity can be considered as an important target in the management of Alzheimer’s disease. The compounds isolated from *C. oxyacantha* were evaluated for cholinesterases inhibitory activity using Ellman’s assay with Galantamine as standard drug. Total of nine (**1–9**) compounds were isolated. Compounds **1** and **2** were isolated for the first time from natural source. Important natural products like β-Sitosterol-3-*O*-β-D-Glucopyranoside (**3**), lupeol (**4**), β-sitosterol (**5**), betulin (**6**), betulinic acid (**7**), oleanolic acid (**8**), and chrysin (**9**) have also been isolated from *C. oxyacantha.* Overall, all the compounds exhibited an overwhelming acetylcholinesterase (AChE) inhibition potential in the range 5.22–44.47 μM. The compound **3** was prominent AChE inhibitor with IC_50_ value of 5.22 μM. Likewise, all the compounds were also potent in butyrylcholinesterase (BChE) inhibitions with IC_50_s of up to 0.55–15.36 μM. All the compounds, except **3**, were selective toward BChE. Mechanism of the inhibition of both the enzymes were further studied by docking procedures using Genetic Optimization for Ligand Docking suit v5.4.1. Furthermore, computational blood brain barrier prediction of the isolated compounds suggest that these are BBB+.

## Introduction

Neurodegenerative disorders affecting a huge number of elder population worldwide (Novakovic et al., 2013). The etiology of neuronal death in these diseases remains inexplicable (Beal, 1995). The onset of these diseases is quite dangerous with gradual progression (Howes et al., 2003). The disorders include Alzheimer’s disease (AD), Huntington’s disease, Parkinson’s disease, amyotrophic lateral sclerosis, and cerebellar degenerations (Beal, 1995). Of these, AD is a chronic neurodegenerative disorder and is the most common type of dementia (Yoshita et al., 2001). AD is supposed to be account for 50–60% of dementia cases in elderly patients (Francis et al., 1999). Major symptoms associated with AD are memory loss, behavioral turbulence and mood disturbance in routine life (Kumar et al., 1998). Several biochemical pathways are followed for the management of AD but one of the most established approach is the inhibition of acetylcholinesterase (AChE) (Zeb et al., 2014; Ahmad et al., 2015; Kamal et al., 2015; Sadiq et al., 2015). The AChE is found in excitable tissues in most erythrocytes and in the placental tissues while butyrylcholinesterase (BChE) is present in nervous system, liver and plasma (Massoulié et al., 1993). In cholinergic synapse, a vital neurotransmitter acetylcholine (ACh) is break down into acetyl group and choline by AChE and BChE (Voet and Voet, 1995). The reduction of ACh concentrations in the hippocampus and cortex of brain can bring a vital biochemical change in AD patients (Jaén et al., 1996). Therefore, a key target in the management of AD is the inhibition of AChE and BChE (Schneider, 1995). Several cholinesterase inhibitors from natural and synthetic sources are available like galantamine, donepezil, rivastigmine, and tacrine (Schulz, 2003). Numerous researchers have also shown that oxidative stress is an early pathogenic event in AD (Sadiq et al., 2015). Therefore, they supplement their anticholinesterase with antioxidants as free radicals scavengers. A constant research is going in the field of AD, which brought several reports from natural product sources and synthetic origin (Ayaz et al., 2014; Sadiq et al., 2015). However, most of these remedies are associated with severe side effects, low efficacy or availability. Therefore, the researchers working on AD are in constant search of novel, safe, effective, and economical origin of drugs.

Recently, galantamine isolated from several species of Amaryllidaceae family including *Galanthus* species, *Leucojum* species, and *Narcissus* species was effectively marketed for the symptomatic relief of AD (Parys, 1998). Galantamine mediate its therapeutic effect via reversible inhibition of acetyl cholinesterase (AChE) and allosterically mediate the action of nicotinic cholinergic receptors (nAChRs). Its selectivity against cholinesterase of different origin is variable. For instance the galantamine selectivity against human erythrocytes (RBCs) based AChE is fifty three times greater than plasma based BChE. Furthermore, galantamine exhibited 10 times lower potency against human brain based AChE as compared to RBCs variant (Harvey, 1995).

*Crataegus oxyacantha*, a flowering shrub of *Rosaceae* family is medicinally used as cardiovascular tonic, anti-hypertensive and agent to induce blood lipid profile (Weihmayr and Ernst, 1996). This plant, also known as hawthorn is an economical and rich source of triterpenic acids, ursolic acid, oleanolic acid, polyphenols like procyanidins, epicatechin, hyperoside, isoquercitrin, chlorogenic acid, and other important organic molecules (Cui et al., 2006). In this piece of research work, we have isolated bioactive compounds from the aerial parts of *C. oxyacantha*. Furthermore, the purified compounds after structure confirmations were subjected to anticholinesterase inhibition assay.

In the current study we have isolated two new (**1** and **2**) and seven (**3–9**) reported compounds from the *C. oxyacantha*. *C. oxyacantha* is herbal supplement and is mainly known for its antioxidant, antimicrobial, anti-inflammatory, gastroprotective, and anti-arrhythmic potentials (Tankanow et al., 2003; Tadic‘ı et al., 2008; Kashyap et al., 2012; Kostić et al., 2012). The compound **3** isolated from *C. oxyacantha*, also called β-sitosterol-3-*O*- β -D-glucopyranoside is majorly reported with antitumor (Guevara et al., 1999), antiprotozoal (Alanís et al., 2003), antimicrobial (Kuete et al., 2007), and as DNA polymerase inhibitor (Mizushina et al., 2006). Lupeol a naturally occurring triterpenoid is recently identified and quantified by HPLC analysis in the hawthorn ethanolic extract (Rezaei-Golmisheh et al., 2015). Lupeol (**4**) has been studied for its possible use in hepatoprotective (Sunitha et al., 2001), anti-inflammatory (Fernández et al., 2001), anticancer (Saleem et al., 2001, 2004), and in inhibition of protein tyrosine phosphatase (Na et al., 2009). An important phytosterol, i.e., β-sitosterol (**5**) was also isolated from *C. oxyacantha*. β-Sitosterol mostly found in vegetable oil, nuts, and avocados has also been reported with clinical trial for it possible use in the management of benign prostatic hyperplasia (Berges et al., 1995). Betulin (**6**) can be easily converted to its structure analog betulinic acid (**7**) and those compounds are reported with identical activities like antimalarial (Alakurtti et al., 2006), anti-inflammatory (Alakurtti et al., 2006), anticancer (Alakurtti et al., 2006), antiviral (Pavlova et al., 2003), and as anti-AIDS (Sun et al., 1998). The oleanolic (**8**) and ursolic acids are famous for their reported activities like hepatoprotective, anti-inflammatory, antihyperlipidemic, antioxidant, and antitumor effects (Liu, 1995). *C. oxyacantha* is a rich source of flavonoids (Li et al., 2009). Chrysin (**9**), naturally occurring flavonoid is majorly reported for its possible use in the management of cancer (Zheng et al., 2003), anxiety (Brown et al., 2007), inflammation (Woo et al., 2005), and in behavioral effects (Zanoli et al., 2000). Based on the literature survey it can be obviously scrutinized that there is no report available on the acetyl or BChE inhibitions of the compounds isolated from *C. oxyacantha*.

## Materials and Methods

### General Information

Column Chromatography was used to purify the compounds using silica gel (E. Merck, 70–300 mesh) and flash silica gel (E. Merck, 230–400 mesh). Thin-layer chromatography (TLC) was carried out pre-coated aluminum sheets (60 F_254_, E. Merck). Purity of the compounds was also checked on the same pre-coated plates visualized under UV light (254 and 366 nm). UV spectra were obtained on Optima SP3000 plus (Japan) using chloroform and methanol as solvent. IR spectra were analyzed on an Elmer Fourier-Transform spectrometer, using KBr plates. ^1^H, ^13^C NMR, and 2D-NMR (HMQC, HMBC, NOSEY, and COSEY) spectra were recorded on a JEOLl ECA 600 (United States) and Bruker AV 500 (Germany) spectrometers. Tetramethylsilane (TMS) was used as an internal standard and Chemical shifts (δ) were expressed in ppm relative to TMS.

### Plant Material

*Crataegus oxyacantha* was collected from local area of Pashtonai (72° 18′ 36″ E, 35° 03′18′ N), KP, Pakistan in June 2013 during flowering season. Plant was identified by Prof. Mahboob Ur Rehman, Govt. Jehanzeb College Swat. Voucher specimen (C-124) was retained for verification purpose in herbarium of the College.

### Extraction and Isolation

Plant twigs were shade dried at room temperature and chopped. Dry powdered plant (22 Kg) was extracted with methanol (3 L × 30 L) at room temperature. The methanolic extract was concentrated under reduced pressure at 50°C using rotary evaporator (R-301, Bucchi) and obtained a gummy extract (1 Kg). The extract was suspended in water and successively partitioned to hexane, dichloromethane, ethyl acetate, and butanol fractions. DCM soluble fraction was subjected to column chromatography over silica gel using *n*-hexane: ethyl acetate and ethyl acetate: methanol as eluting solvent system which yielded nine daughter fractions (S1–S9). On repeated column chromatography of these nine resulted in two new (**1–2)** seven (**3–9**) known compounds.

### [2-(3, 4-Dimethoxyphenyl)-2-Methoxyethanol] (1)

Slightly greenish in color_,_ IR (KBr) max: 3425, 1570, 3450, and 1245 cm^-1^; EI-MS *m/z*: 212 [M+] (calcd. 212.1049 for C_11_H_16_O_4_); ^1^H-NMR (DMSO, 300 MHz) δ: 6.87 (1H, s, H-2), 6.71 (1H, m, H-5), 6.71 (1H, m, H-6), 4.60 (1H, d, *J =* 3.9Hz, H-7), 4.12 (2H, t, *J* = 2.9Hz, H-8), 3.71 (3H, s, Me-9), 3.70 (3H, s, Me-10), 3.01 (3H, s, Me-11).

### 3-Hydroxy-1-(4-Hydroxy-3-Methoxyphenyl) Propan-1-One (2)

Amorphous solid compound; IR (KBr) max: 3420, 1685, 1580, 3460, and 1240 cm^-1^; EI-MS *m/z*: 194 [M+] (calcd. 194.0943 for C_11_H_14_O_3_); ^1^H-NMR (DMSO, 300 MHz) δ: 3.04 (2H, t, *J* = 6.3Hz, H-2), 3.74 (2H. t, *J* = 6.3Hz, H-3), 3.81 (3H, s, Me-4), 7.43 (1H, d, *J* = 1.2Hz, H-2′), 6.85 (1H, d, *J* = 8.1, H-5′), 7.50 (1H, dd, *J* = 8.1, 1.2Hz, H-6′).

### β-Sitosterol-3-*O*-β-D-Glucopyranoside (3)

White amorphous solid; IR *ν*_max_ (KBr): 3452, 1648, 1379 and 1065 cm^-1^; EI-MS: *m*/*z* 414.0000 (calcd. for [C_29_H4_50_O]^+^); ^1^H-NMR (DMSO, 300 MHz) δ: 1.20 (2H, m, H-1), 1.66 (2H, s, H-20), 3.51 (1H, m, H-3), 2.21 (2H, m, H-4), 5.35 (1H, m, H-6), 1.40 (2H, m, H-7), 1.44 (1H, m, H-8), 1.56 (1H, m H-9), 1.40 (2H, s, H-11), 1.41 (2H, s, H-12), 1.40 (1H, m, H-14)1.42 (2H, m, H-15), 1.86 (2H, s, H-16), 1.48 (2H, m, H-17), 0.64 (3H, s, Me-18), 1.0 (3H, m, Me-19), 1.66 (1H, m, H-20), 0.92 (3H, d, Me-21), 1.68 (2H, s, H-22), 0.83 (2H, s, H-23), 0.82 (1H, s, H-24), 1.2 (1H, m, H-25), 0.82 (3H, d, *J* = 6.51 Hz, Me-26), 0.81 (3H, d, *J* = 6.51 Hz, Me-27), 1.34 (2H, m, H-28), 0.84 (3H, t, *J* = 6.91 Hz, Me-29), 4.57 (1H, d, *J* = 7.51 Hz, H-1′), 3.14 (1H, m, H-2′), 3.21 (1H, m, H-3′), 3.24 (1H, m, H-4′), 3.35 (1H, m, H-5′), 3.85 (2H, dd, *J* = 11.8 Hz, H-6′).

### Lupeol (4)

White powder; FT-IR (neat) max: 3406, 1645, 1495, 1381, 1183, 1104, 1039, 985, 940 cm^-1^. Molecular formula: C_30_H_50_O; EI-MS *m/z*: 426 [M+] (calcd. 426.0000 for C_30_H_50_O); EI-MS *m/z* (rel. int.) (%): 426 (55.45), 393 (3.71), 315 (13.57), 257 (10.04), 234 (18.16), 189 (68.11), 161 (30.98), 135 (63.03); ^1^H-NMR (CDCl_3_, 300 MHz) δ: 1.69 (2H, dd, *J* = 6.12Hz, H-1), 1.37 (2H, m, H-2), 3.16 (1H, dd, *J* = 11.46 Hz, 4.8 Hz, H-3), 0.66 (1H, m, H-5), 1.37 (2H, m, H-6), 1.37 (2H, m, H-7), 1.24, 1.27 (1H, m, H-9), 1.55, 1.59 (2H, m, H-11), 1.66 (2H, m, H-12), 0.89, 1.69 (1H, m, H-13), 1.55 (2H, d, H-15), 1.37 (2H, m, H-16), 1.32 (1H, d, H-18), 2.37 (1H, m, H-19), 1.24 (2H, q, H-21), 1.18 (2H, m, H-22), 0.95 (3H, s, Me-23), 0.81 (2H, s, H-24), 1.01 (3H, s, Me-25), 0.74 (3H, s, Me-26), 0.93(3H, s, Me-27), 0.77 (3H, s, Me-28), 4.46 (2H, m, H-29), 1.66 (3H, s, Me-30).

### β-Sitosterol (5)

White powder; IR (KBr) max: 3406, 2905, and 1640 cm^-1^; Molecular formula C_29_H_50_O; EI-MS: *m/z* 414 [M+] (calcd. 414.0000 for C_29_H_50_O); ^1^H-NMR (DMSO_,_ 300 MHz) δ: 1.07 (2H, dd, *J* = 6.12 Hz, H-1), 1.23, 1.48 (2H, m, H-2), 3.52 (2H, m, H-3), 1.98 (2H, t, H-4), 5.33 (1H, d, H-6), 1.44 (2H, m, H-7), 1.42 (1H, m, H-8), 0.84 (1H, m, H-9), 1.49, 1.47 (1H, m, H-11), 2.19 (2H, d, *J* = 2.1, H-12), 0.96 (1H, m, H-14), 1.56 (2H, m, H-15), 1.23 (3H, m, Me-16), 1.07 (1H, d, *J* = 7.8, H-17), 0.66 (3H, s, Me-18), 0.99 (3H, s, Me-19), 1.35 (1H, m, H-20), 0.90 (3H, d, *J* = 5.26Hz, Me-21), 1.27 (2H, m, H-22), 1.14 (2H, m, H-23), 0.92 (1H, m, H-24), 1.26 (1H, m, H-25), 0.79 (3H, d, *J* = 6.8 Hz, Me-26), 0.81 (3H, d, *J* = 5.58 Hz, Me-27), 1.22 (2H, m, H-28), 0.82 (3H, t, *J =* 7.5 Hz, Me-3).

### 3β, 28-Dihydroxylup-20(29)-ene (Betulin) (6)

White amorphous powder; IR (KBr) ý_max_: 3400, 1603.4 cm^-1^; EI-MS m/z 442 [calcd. 442.0000 for M^+^, C_30_H_50_O_2_]; ^1^H-NMR (MeOD_,_ 300 MHz) δ: 1.44 (2H, m, H-1), 1.44 (2H, m, H-2), 4.01 (1H, dd, *J* = 10.8 Hz, H-3), 1.40 (1H, m, H-5), 1.42 (2H, m, H-6), 1.44 (2H, m, H-7), 1.39 (1H, m, H-9), 1.42 (2H, m, H-11), 1.42 (2H, m, H-12), 1.40 (1H, m, H-13), 1.44 (2H, m, H-15), 1.44 (2H, m, H-16), 1.42 (1H, m, H-18), 2.39 (1H, m, H-19), 1.39 (2H, m, H-21), 1.30 (2H, m, H-22), 0.94 (3H, s, Me-23), 0.80 (3H, s, Me-24), 0.74 (3H, s, Me-25), 1.06 (3H, s, Me-26), 1.00 (3H, s, Me-27), 3.40 (2H, m, H-28), 4.66 (3H, s, Me-29), 1.66 (3H, s, Me-30).

### 3β-Dihydroxylup-20(29)-ene-28-oic Acid (Betulinic Acid) (7)

White amorphous powder; IR (KBr) ý_max_: 3500, 1700, 1625 cm^-1^; EI-MS m/z 442 [M^+^, C_30_H_48_O_3_]; ^1^H-NMR (DMSO_,_ 300 MHz) δ: 1.44 (2H, m, H-1), 1.44 (2H, m, H-2), 3.47 (1H, dd, *J* = 10.2 Hz, H-3), 1.39 (1H, m, H-5), 1.41 (2H, m, H-6), 1.44 (2H, m, H-7), 1.40 (1H, m, H-9), 1.41 (2H, m, H-11), 1.41 (2H, m, H-12), 1.40 (1H, m, H-13), 1.44 (2H, m, H-15), 1.87 (2H, m, H-16), 1.71 (1H, m, H-18), 3.0 (1H, t, *J* = 10.2, 5,5 Hz, H-19), 1.47 (2H, m, H-21), 1.72 (2H, m, H-22), 0.82 (3H, s, Me-23), 0.95 (3H, s, Me-24), 0.81 (3H, s, Me-25), 0.95 (3H, s, Me-26), 0.93 (3H, s, Me-27), 4.66 (3H, s, Me-29), 1.68 (3H, s, Me-30).

### 3β-Hydroxyurs-12-en-28-oic Acid (Oleanolic Acid) (8)

Colorless crystals (CHCl_3_–MeOH); IR (KBr) ý_max_: 3450 cm^-1^, 1700 cm^-1^; EI-MS m/z 456 [M^+^, C_30_H_48_O_3_]; ^1^H-NMR (DMSO_,_ 300 MHz) δ: 1.43 (2H, m, H-1), 1.61 (2H, m, H-2), 3.44 (1H, dd, *J* = 11.2, 5.5 Hz, H-3), 1.37 (1H, m, H-5), 1.38 (2H, m, H-6), 1.43 (2H, m, H-7), 1.44 (1H, m, H-9), 1.90 (2H, m, H-11), 5.50 (2H, Brs, H-12), 1.24 (2H, m, H-15), 1.47 (2H, m, H-16), 2.53 (1H, d, *J* = 12.0 Hz, H-18), 1.34 (2H, m, H-19), 1.43 (2H, m, H-21), 1.87 (2H, m, H-22), 1.24 (3H, s, Me-23), 1.01 (3H, s, Me-24), 0.88 (3H, s, Me-25), 1.03 (3H, s, Me-26), 1.30 (3H, s, Me-27), 0.94 (3H, d, *J* = 6.2 Hz, Me-29), 1.02 (3H, s, Me-30).

### 5,7-Dihydroxyflavone (Chrysin) (9)

Slightly yellow Crystals; IR νmax (KBr): 3406, 1656, 3090, 1600, 1571, 1500 cm^-1^; EI-MS: m/z 254 [calcd. 254.0579 for C_15_H_10_O_4_]; ^1^H-NMR (DMSO_,_ 300 MHz) δ: 6.76 (1H, s, H-3), 6.28 (1H, d, *J* = 1.9 Hz, H-6), 6.57 (1H, d, *J* = 1.9 Hz, H-8), 8.05 (1H, dd, *J* = 8.1 Hz, H-2′), 7.58 (1H, m, H-3′), 7.60 (1H, m, H-4′), 7.58 (1H, m, H-5′), 8.05 (1H, dd, *J* = 8.1 Hz, H-6′).

### *In Vitro* Cholinesterase Inhibitory Assays

*In vitro* cholinesterase inhibitory potentials of our test compounds were evaluated using Ellmans assay (Ellman et al., 1961; Ayaz et al., 2015). Basic principle of this procedure is reliant on catalytic degradation of substrates including acetylthiocholine iodide and butyrylthiocholine iodide by the respective enzymes AChE and BChE to form 5-thio-2-nitrobenzoate anion which is subsequently complexed with DTNB to form a yellow color compound. This yellow compound is UV detectable and is quantified with the passage of time with and without the impact of inhibitor agent. Briefly, from each enzyme solution, 5 μl were added to wells of micro plate and 5 μl DTNB was added to it. The resultant mixture was incubated for 15 min at 30°C in water bath followed by addition of 5 μl substrate solution. Finally, absorbance was measured at 412 nm using micro plate reader. Control contains all solutions except inhibitor. Change in absorbance was recorded along with reaction time. At the end, Enzymes and enzymes inhibitory activities by control and our test samples were calculated from the rate of absorption with change in time as (V = Δ Abs /Δ t) and Enzyme inhibition as; Percent Enzyme activity = 100 × V/V_Max_.

Where, V_Max_ is enzyme activity in the absence of inhibitor agent.

### Molecular Modeling

The molecular construction of isolated compounds were performed using ChemBioDraw Ultra 14 suite (PerkinElmer Inc.) and converted into 3D conformations by ChemBio3D (Mills, 2006). Molecular docking simulations of isolated compounds were carried out using GOLD (Genetic Optimization for Ligand Docking) (version 5.4.1) (Verdonk et al., 2003) software, with goldscore_p450_csd template. Goldscore was selected as a fitness function. Docking search area was sphere of radius 6 Å. Crystal structures of the enzymes, AChE (PDB: 1EVE) and BChE (PDB: PDB Code 1P0I) used for protein–ligand interactions, were retrieved from Protein Data Bank (PDB). The target proteins were prepared by the addition of hydrogen, removal of water and the removal of cocrystallized ligands. All other parameters were used with the default settings. The ligand-bound sites of the enzymes were used as possible binding sites to analyze the potential binding of isolated compounds. Co-crystallized ligands (reference ligands) were also re-docked to compare the results for accuracy. 3D images were taken using UCSF Chimera 1.11 software (Pettersen et al., 2004), while 2D images were taken using (Biovia, 2016).

## Results

**FIGURE 1:**
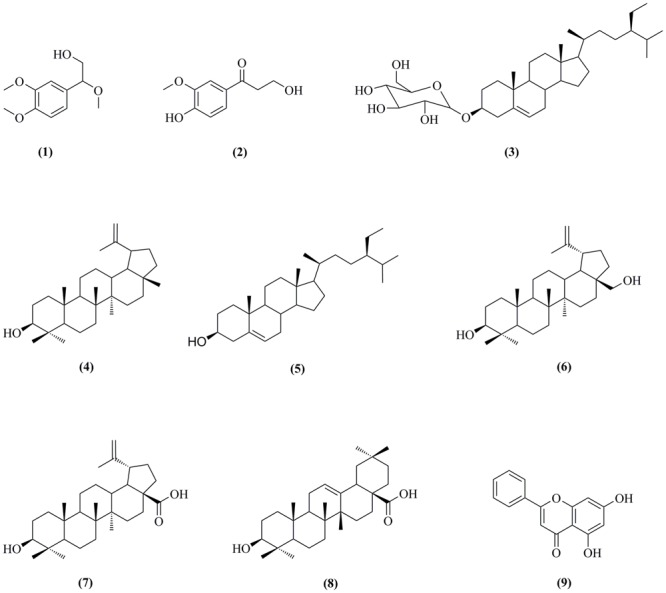
Isolated organic molecules from dichloromethane fraction of aerial parts of *Crataegus oxyacantha.*

The *C. oxyacantha* was collected from the district Swat of Pakistan. The crude extract of the plant was obtained using its aerial parts. Based on the initial screening, TLC analysis and quantity of the dichloromethane fraction (80 g), this fraction was selected for isolation of bioactive among other solvent fractions. Initially, the TLC analyzed DCM fraction was subjected to a major chromatography which resulted in semi-purified organic molecules. Then by using a small scale column chromatography for all of individual samples resulted in isolation of nine organic molecules (compounds **1–9, Figure 1**). To the best of our literature survey we claim that compounds **1** [2-(3, 4-dimethoxyphenyl)-2-methoxyethanol] and **2** [3-hydroxy-1-(4-hydroxy-3-methoxyphenyl) propan-1-one] were obtained for the first time from the natural sources. The remaining seven compounds are pre-existing important natural products including β-Sitosterol-3-*O*-β-D-Glucopyranoside (**3**), lupeol (**4**), β-sitosterol (**5**), betulin (**6**), betulinic acid (**7**), oleanolic acid (**8**), and chrysin (**9**). All the isolated compounds are very important moieties in natural products.

### *In Vitro* Pharmacology

Initially, we evaluated the dichloromethane fraction for its possible potential of inhibition the acetyl and BChE s. After getting encouraging results from the initial screening/extract, pure compounds (**1–9**) were subjected to the acetyl and BChE e inhibitions as shown in **Table 1**.

**Table 1:** Results of *in vitro* cholinesterase inhibitory assays on the isolated compounds.

l Compound	Concentration (μg/ml)	% AChE inhibition	IC_50_ (μM ± SEM)	% BChE inhibition	IC_50_ (μM ± SEM)	AChE^a^ selectivity	BChE^b^ selectivity
l**1**	250	97.32 ± 1.06		90.65 ± 1.34			
l	125	95.70 ± 1.25	23.2 ± 1.02	87.44 ± 0.58	2.41 ± 0.09	0.10	9.62
l	62.5	89.65 ± 1.47		84.28 ± 2.19			
l**2**	250	93.05 ± 0.75		95.76 ± 0.71			
l	125	92.74 ± 1.29	25.90 ± 1.35	91.23 ± 1.83	15.36 ± 1.01	0.59	1.69
l	62.5	83.74 ± 0.68		87.56 ± 1.06			
l**3**	250	95.70 ± 1,60		94.84 ± 0.30			
l	125	91.90 ± 0.43	5.22 ± 0.59	88.94 ± 1.13	11.83 ± 0.93	2.26	0.47
l	62.5	87.90 ± 0.52		83.03 ± 0.35			
l**4**	250	93.73 ± 0.78		91.42 ± 0.43			
l	125	90.37 ± 1.65	36.31 ± 1.89	89.91 ± 0.88	1.31 ± 0.12	0.04	27.71
l	62.5	77.87 ± 0.26		85.80 ± 1.50			
l**5**	250	91.33 ± 0.49		92.58 ± 1.12			
l	125	86.67 ± 0.89	14.57 ± 0.85	90.00 ± 0.00	0.56 ± 0.03	0.04	26.62
l	62.5	80.00 ± 0.00		87.08 ± 0.47			
l**6**	250	96.13 ± 0.20		95.85 ± 2.25			
l	125	94.03 ± 0.48	23.98 ± 1.10	93.72 ± 1.01	25.63 ± 1.25	1.06	0.93
l	62.5	84.00 ± 1.15		82.45 ± 0.90			
l**7**	250	95.70 ± 1.60		94.10 ± 0.60			
l	125	91.90 ± 0.43	44.47 ± 1.73	90.25 ± 1.40	3.48 ± 0.15	0.07	12.78
l	62.5	77.70 ± 1.25		87.22 ± 1.28			
l**8**	250	95.05 ± 0.75		93.08 ± 1.04			
l	125	92.00 ± 0.98	38.83 ± 1.68	89.93 ± 0.67	5.16 ± 0.13	0.13	7.52
l	62.5	79.62 ± 1.67		85.59 ± 3.28			
l**9**	250	93.67 ± 0.88		85.00 ± 0.00			
l	125	90.76 ± 0.61	32.30 ± 1.80	82.00 ± 1.15	0.63 ± 0.06	0.02	51.27
l	62.5	81.20 ± 0.23		80.90 ± 1.90			
l**Galantamine**	250	96.62 ± 1.67		95.33 ± 1.09			
l	125	92.91 ± 1.30	8.0 ± 0.12	92.50 ± 1.04	10.0 ± 0.10	1.25	0.8
l	62.5	89.87 ± 1.27		87.83 ± 1.01			

*^a^Selectivity Index for AChE = IC_*50*_ ratio (BChE/AChE). ^b^Selectivity Index for BChE = IC_*50*_ ratio (AChE/BChE).*

In AChE inhibition assay, almost all of the compounds exhibited considerable results with IC_50_ values of greater potency as shown in **Table 1**. In AChE inhibitory assays, Compounds 1, 6, 3, 7, 8 exhibited highest AChE inhibitory activities displaying 97.32 ± 1.06, 96.13 ± 0.20, 95.70 ± 1.60, 95.70 ± 1.60, and 95.05 ± 0.75% inhibitions at 250 μg/ml, respectively. The AChE inhibitory activities of these samples were very much comparable with the galantamine at the same concentrations **Table 2**. In BChE inhibition assay, 2, 3, 6, and 7 showed highest inhibitory activity with inhibitions of 95.76 ± 0.71, 94.84 ± 0.30, 95.85 ± 2.25, 94.10 ± 0.60, respectively. Percent inhibitions of our test compounds were more against AChE in comparison to BChE. Among the entire compound **3** showed the most prominent inhibition with IC_50_ value of 5.22 μM. At the same three concentrations the standard drug Galantamine was observed with IC_50_ = 8.0 μM. Newly isolated compounds **1, 2** displayed moderate activities with IC_50_ values of 23.2 and 25.90 μM, respectively. The order of potency of the remaining compounds was **5** > **6** > **9** > **8** > **7** (**Table 1**).

**Table 2:** Physico-chemical descriptors of the isolated compounds **(1–9).**

Compound no.	MW	LogP	HBD	HBA	nROT	tPSA	BBB
1	212.25	0.61	1	4	5	47.9	+ve
2	196.2	0.14	2	4	4	66.76	+ve
3	576.86	5.48	4	6	9	99.38	-ve
4	426.73	7.73	1	1	1	20.23	+ve
5	412.75	9.29	2	0	6	0.00	+ve
6	442.73	6.43	2	2	2	40.46	+ve
7	456.71	6.96	2	3	2	57.53	+ve
8	456.71	7.02	2	3	1	57.53	+ve
9	254.24	2.4	2	4	1	70.67	+ve

A summary of the BChE inhibitions of compounds **1–9** is also shown in **Table 1**. In BChE assay all the compounds achieve lower IC_50_ values than that of the standard drug Galantamine. With exception of compounds **3** and **6**, all the other isolated compounds exhibited excellent selectivity toward BChE (**Table 1**). Compounds **5** and **9** have shown the BChE inhibition in nano-molar range (IC_50_ = 0.55 and 0.63 μM, respectively). Compound **6** exhibited very little activity (IC_50_ = 25.63 μM). In the current study galantamine selectivity was definitely greater for AChE (56%) more than BChE as given in **Table 1**.

### Molecular Modeling Studies

**FIGURE 2:**
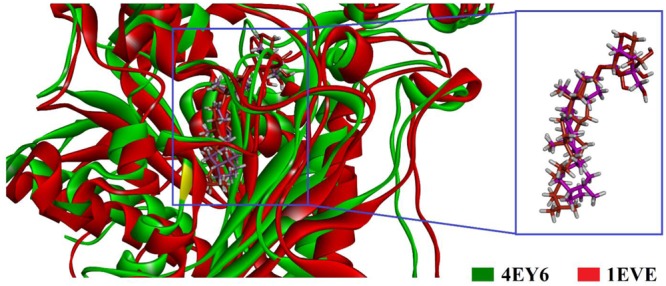
Superimposed ribbon diagram of the top-scoring docking pose for isolated compound **3**-*Tc*AChE (PDB ID 1EVE, red ribbons) and **3**-*h*AChE complex (PDB ID 4EY6, green ribbon).

To gain insight into the mechanism of AChE and BChE inhibition, binding modes of the isolated compounds were explored by GOLD suit v5.4.1. There are a plenty of X-ray crystal structures of AChE in PDB like *Drosophila melanogaster, Electrophorus electricus* (eel), *Torpedo californica* (*Tc*AChE), human (*h*AChE) and mouse in apo or with co-crystallized ligand. These crystal structures can be used for the designing of new AchE inhibitors. In the current study, we tried to present the possible mechanism of action of the isolated compounds. For this purpose, X-ray crystal structure of TcAChE and hAChE also studied. The X-ray crystallographic structure of *Tc*AChE (PDB Code 1EVE) and *h*AChE (PDB Code 4EY6) in complex with donepezil and galantamine were used as enzyme structures. Superposition of the docked poses of the most active compound **3** into the binding pockets of *Tc*AChE and *h*AChE is shown in **Figure 2A**. The binding mode analysis of isolated compound **3**-*Tc*AChE and **3**-*h*AChE complex shows that the compound **3** have same binding orientation in binding pocket of *Tc*AChE and *h*AChE.

**FIGURE 3:**
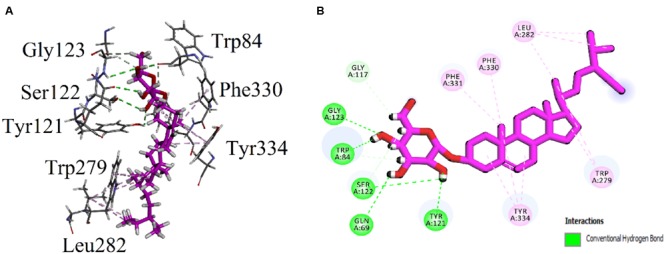
**(A)** Stereoview of the docking pose of β-Sitosterol-3-*O*-β-D-Glucopyranoside **(3)**, (blue color stick model) in the binding pocket of AChE (1EVE). **(B)** Two dimensional (2D) interactions of β-Sitosterol-3-*O*-β-D-Glucopyranoside **(3).** Conventional hydrogen bonding interactions are depicted in green and hydrophobic interactions in light pink (Prepared by using Discovery Studio Visualizer).

**FIGURE 4:**
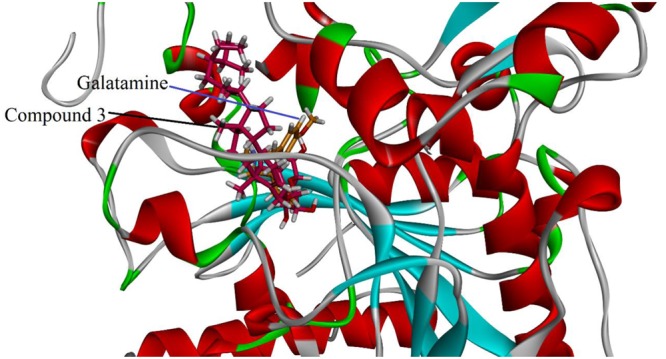
Putative binding mode of **3** in the binding cavity of human AchE (PDB Code 4EY6). The structure reveals that compound **3** is embedded in the binding pocket of galantamine.

Visual inspection of all the best docked pose of the most active compound **3** in the active site of 1EVE revealed that hydrocarbon chain is embedded into the hydrophobic slot formed by Trp279 and Leu282. Tetrahydropyran ring formed five conventional hydrogen bonding interactions with Gln69, Trp84, Tyr121, Ser122, and Gly123. Tetracyclic ring structure is sandwiched between bottleneck residue Phe330 and Tyr334 (**Figure 3**). Isolated compound **3** showed highest Gold fitness score (79.9353). To understand the mechanism, we also docked compound **3** into the binding *h*AChE co-crystallized with galantamine. **Figure 4**, reveals that it is embedded into the binding site of galantamine.

**FIGURE 5:**
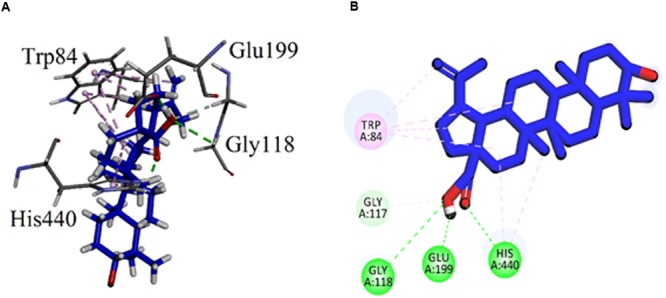
**(A)** Stereoview of the docking pose of Betulinic acid (**7**, blue color stick model) in the binding pocket of AChE (1EVE). **(B)** 2D interactions of betulinic acid **(7)**.

The binding mode of the compound **7** (Betulinic acid), the least active compound with IC_50_ value of 44.47 μM, was also analyzed. The carboxylic group of compound **7** formed conventional hydrogen bonding interactions with the catalytic triad residue (His440) and oxyanion hole residue (Gly118). It also established with the acidic residue Glu199 near the base of the gorge (**Figure 5**). The Gold fitness score for **7** is 47.8194.

**FIGURE 6:**
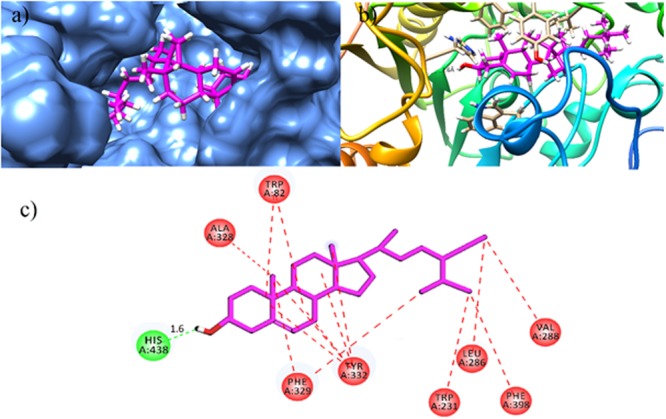
Docked binding pose of the most active isolated compound β-sitosterol **(5**) in the binding pocket of BChE (magenta color stick model); Surface diagram **(a)** and Ribbon **(b)** as rendered by Chimera 1.11.2rc. **(c)** 2D interactions of **(5).** Conventional hydrogen bonding interactions are depicted in green and hydrophobic interactions in red (Prepared by using Discovery Studio Visualizer).

For gaining insight into the mechanism of BChE inhibition, the X-ray crystallographic structure of human BChE (PDB Code 1P0I) was used as enzyme structure. The environment of the active site BChE gorge is hydrophobic due to presence of aromatic and aliphatic amino acid residues. This hydrophobic patch consists of Trp82, Tyr128, Ala328, and Phe329. The acyl pocket contains leu286 and Val288. The amino acid residues in wall of the gorge are Trp114, Trp231, Tyr332, Trp430 and Tyr440. Structural analysis has revealed that all the isolated compounds (**1–9**) contain both hydrophobic alkyl chains as well as hydrophilic features. The most active BChE inhibitor compound **5** (β-sitosterol) is a hydrophobic molecule as indicated by its logP value (9.29, **Table 2**). Docked binding pose of β-sitosterol in the binding pocket of BChE is shown in **Figures 6a,b**. β-Sitosterol (**5**) is located in hydrophobic patch and forms mainly hydrophobic interactions with Trp82, Trp231, Leu286, Val288, Ala328, Phe329, and Tyr332 (**Figure 6c**). β-Sitosterol-BChE complex also stabilized by the formation of hydrogen bonding interactions between one of the residue of catalytic triad (His438) and hydroxyl group of β-sitosterol at the distance of 1.6 Å.

#### Preliminary *In Silico* Pharmacokinetics

Poor pharmacokinetic properties are the main cause of failure of the drugs or lead compounds to enter the market. Prediction of *in silico* ADMET studies (prediction of the *in vivo* pharmacokinetics) as early as possible in the drug discovery process has now become imperative to develop drugs with improve oral bioavailability, reduced toxicity, and adverse side effects.

Lipinsiki criteria for oral absorption was computed by using Molinspiration online software. The analysis of the Lipinsiki’s descriptors tabulated in **Table 2** indicated compound **3** have molecular weight >500. Isolated compounds **3–8** violated the Lipinski’s Ro5 due to their logP value > 5 (**Table 2**, highlighted in red). Compound **3** is predicted to be the most lipophilic compound due to its high value of logP. Polar surface area is another important criteria for the determination of BBB penetration. According to Waterbeemd the cutoff value is 90 Å^2^ or less []. Compound **3** violates the criteria for cutoff value of tPSA (99.38). Number of rotatable bonds (nROT) is an additional property that measures the flexibility of the molecule. It can also be used as a filter in the ADME predictions. The drugs that penetrate BBB usually reported to have fewer nROT counts.

## Discussion

This study was designed to further validate the medicinal uses of *C. oxyacantha* based on anticholinesterase activity and computational studies of pure principles from the plant. In terms of enzyme ihibition, dichloromethane soluble fraction with highest activity was separated into nine compounds (**1–9**). This work is a first report on enzyme inhibitory potential of *C. oxyacantha*.

As shown in **Figure 1**, the isolated compounds (**1–9**) contain both hydrophobic alkyl chains as well as hydrophilic features. In AChE activity, we have demonstrated that compound **3** is the most active one with IC_50_ value of 5.22 μM in comparison to the standard drug Galantamine with IC_50_ = 8.0 μM. From molecular docking point of view, for the most active compound **3** (Gold fitness score = 79.9353), the hydrocarbon side chain and sugar moiety are the best features needed for the enzyme activity (**Figure 2**). For compound **7** which is the least active (IC_50_ = 44.47 μM), binding modes have been recorded for carboxylic acid group (**Figure 3**). Amongst the compounds, β-sitosterol (**5**) was found highly active in BChE inhibition with IC_50_ value of 0.56 μM. This activity can be attributed to the docking positions of side chain and hydroxyl group in compound **5** (**Figure 4**).

We assessed the *in silico* pharmacokinetic profile of the isolated natural products. Although most of these isolated compounds are reported in literature, the aim of this study is to predict their blood brain barrier (BBB) penetration by detailed understanding of physicochemical descriptors (**Table 2**). Targeting the central nervous system is a challenge for drug designing chemists due to the tight junctions of the BBB epithelial cells (Pajouhesh and Lenz, 2005). Therefore, BBB penetration is required by the drug candidates targeting AChE. For this purpose, online software admetSAR was used to predict whether the isolated compounds can cross blood brain barrier (BBB+) or not (BBB). However, with exception of compound **3**, all isolated compounds are predicted to cross blood brain barrier (i.e., BBB+).

## Conclusion

Herein, we claim that *C. oxyacantha* is a rich source of neurologically potent organic molecules. The isolated compounds from *C. oxyacantha* are evaluated for the first time for its possible use in acetyl and BChE inhibitions. All the isolated compounds showed an overwhelming acetyl and BChE inhibitions. There is good agreement found between *in vitro* experimental results and the docking scores.

## Author Contributions

MA and AL wrote the manuscript and also did spectral analyses and literature studies. SM performed plant collection and isolation of compounds. AK, UF, and UR performed computational studies. MS facilitated isolation of compounds and their mass and NMR spectra. FU, AS, MAy, and MAl were involved in doing biological activities. MAh did the final confirmation of the elucidated molecular structures.

## Conflict of Interest Statement

The authors declare that the research was conducted in the absence of any commercial or financial relationships that could be construed as a potential conflict of interest.
